# Epidemiological and Microbiome Characterization of Black Tooth Stain in Preschool Children

**DOI:** 10.3389/fped.2022.751361

**Published:** 2022-01-26

**Authors:** Yu Zhang, Rui Yu, Jing-Yu Zhan, Gui-Zhi Cao, Xi-Ping Feng, Xi Chen

**Affiliations:** ^1^Shanghai Key Laboratory of Stomatology, Department of Preventive Dentistry, Shanghai Ninth People's Hospital, College of Stomatology, Shanghai Jiao Tong University School of Medicine, National Clinical Research Center for Oral Diseases, Shanghai Research Institute of Stomatology, Shanghai, China; ^2^College of Veterinary Medicine, Henan Agricultural University, Zhengzhou, China

**Keywords:** preschool children, black tooth stain, plaque, saliva, flora microorganisms, epidemiological study

## Abstract

**Objective:**

To assess the epidemiologic attributes and microbial variations associated with extrinsic black tooth stain (BTS) among Chinese preschool children.

**Methods:**

This cross-sectional study included 250 preschool children (3–4 years) from three kindergartens in Shanghai, China. Following clinical examination, and using a case-control design, saliva and dental plaque specimens were collected from caries-free participants with (*n* = 21, BTS group) and without (*n* = 48, control group) BTS. The chi-square test and logistic regression model were used to evaluate factors associated with BTS. 16S rRNA sequencing were used to characterize the associated microbial communities.

**Results:**

BTS was detected in 12.4% of participants, with a mean of 13.7 black-stained teeth. Participants with BTS had a lower caries burden and better oral hygiene (*P* = 0.003). Children with less frequent intake of marmalade or honey (*P* = 0.033) and regular application of fluoride (*P* = 0.007) had a lower likelihood of having BTS. Microbiota analysis revealed 14 phyla, 35 classes, 63 orders, 113 families, 221 genera, 452 species, and 1,771 operational taxonomic units (OTUs). In terms of microbial diversity, no significant differences were observed in the saliva of the two groups (*P* > 0.05). Dental plaque from the BTS group exhibited higher OTU richness but lower evenness than that from the control group (Chao *P* = 0.006, Shannon *P* = 0.007, respectively) and showed a significant difference in β diversity (*P* = 0.002). The microbiome in the two groups was characterized by various microbial biomarkers, such as *Pseudomonas fluorescens, Leptotrichia sp._HMT_212, Actinomyces sp._HMT_169*, and *Aggregatibacter sp._HMT_898* in plaques from the BTS group. Functional analysis of the microbial species suggested the existence of a hyperactive metabolic state on teeth surfaces with BTS plaques and revealed that ferric iron, the iron complex transport system, and the iron (III) transport system were more abundant in BTS plaque samples.

**Conclusions:**

This study provides insights into the epidemiologic and microbial features of BTS in preschool children. The microbiome in BTS is characterized by various microbial biomarkers, which can serve as indicators for BTS diagnosis and prognosis.

## Introduction

Black tooth stain (BTS) is a common condition characterized by an extrinsic discoloration of teeth due to the deposition of bacterial plaque enriched in calcium phosphate minerals and insoluble iron-containing compounds (1, 2). BTS is clinically diagnosed based on the deposition of dark-colored lines or dots parallel to the gingival margin in the cervical half of the crown (3, 4). Due to the high mineral content, conventional tooth brushing is unlikely to remove BTS, and BTS has a tendency to reform after professional scaling and debridement (5).

BTS affects primary and permanent dentition and is equally prevalent (1–20%) in both genders (5, 6). The black pigmentation is caused by the formation of ferric compounds (such as ferric sulfide) as a result of interactions between hydrogen sulfide (produced by periodontal microorganisms) and iron present in saliva or gingival fluid (7). Therefore, oral microorganisms, particularly chromogenic bacteria (such as *Actinomyces* and *Prevotella*) are associated with the formation of black pigments and BTS in primary dentition (8–10). Heinrich-Weltzien et al. analyzed the compositions of bacterial plaque in patients with BTS using real-time polymerase chain reaction (PCR),and reported that specimens from the BTS group contained increased numbers of *Actinomyces naeslundii* and reduced numbers of *Lactobacillus spp*. and *Fusobacterium nucleatum* (11). A recent study reported the relative abundance of *Pseudopropionibacterium, Actinomyces, Rothia*, and *Cardiobacterium* was higher and that of *Porphyromonas* was lower in the BTS group (4). Although these findings clearly indicate an association between BTS and altered oral microbiota, the basic microbial features and association between the microbial assemblies and BTS are not fully understood. Therefore, to identify the microorganisms underlying the formation of BTS, the interactions between the complex oral environment and microbial flora require further investigations.

BTS can lead to aesthetic, social, and psychological issues in children. Although BTS has significant public health and clinical research implications, the literature and epidemiological data about the deposition of black stains on primary dentition is limited. In addition, various studies reporting the prevalence of BTS among children mainly investigated children aged 6–12 years old or adolescents (11–13). The current literature reporting the prevalence, microbiota, and risk factors of BTS in preschool children is scarce.

Next-generation sequencing (NGS) (14) is an established technology that has empowered researchers to investigate the microorganisms associated with BTS at the molecular level. This technique has facilitated better understanding of the micro-ecological alterations associated with BTS (15). Using NGS, Li et al. discovered that certain bacterial species such as *Leptotrichia* and *Fusobacterium* may contribute to the production of BTS. Furthermore, three bacterial genera (*Clostridiaceae, Peptostreptococcus*, and *Clostridium*) were found to be related to the co-occurrence of black extrinsic discoloration and dental caries (15).

The aim of the present study was to assess the epidemiologic features and microbial variations associated with extrinsic BTS among preschool children aged 3–4 years in Shanghai, People's Republic of China. We used 16S rRNA sequencing to analyze the microbiome of saliva, supragingival plaque, and pigment spots on tooth surface specimens from preschool children with and without BTS.

## Materials and Methods

### Subjects and Study Design

The present epidemiological study included preschool children (3–4 years) from three kindergartens in Shanghai in three randomly selected districts (two in the suburbs and one in the central district) in 2018. Kindergartens were selected using a multi-stage cluster systematic random sampling method according to the list of kindergartens compiled by the Shanghai Education Commission in Shanghai, People's Republic of China. The ratio of public and private kindergartens was also taken into consideration in the sampling process. The required sample size was calculated with the following formula:

n≥t^2^p(1–p)/d^2^

where t is the confidence level at 95%, p is the estimated prevalence of BTS (based on a previous pilot survey, unpublished data, 15% prevalence), and d is the margin of error. The calculated sample size was ≥ 204.

Consent to participate in the study was requested from the parents/guardians of all children (3–4 years) registered at the selected kindergartens. The parents/guardians signed an informed consent form allowing us to proceed with the clinical oral examination and sample collection of their children. The study was performed with ethical approval from the institutional Ethical Committee of the Ninth People's Hospital, School of Medicine, Shanghai Jiao Tong University, Shanghai, China (Ref No. 2015135). This study conformed to the STROBE guidelines and all procedures were performed in accordance with the Declaration of Helsinki.

The inclusion criteria were as follows: (1) systemically healthy; (2) no antibiotic intake in the previous 3 months. Children who were absent or uncooperative on the day of the investigation and those who failed to provide informed consent were excluded.

To obtain information regarding the associated factors for BTS, all participants' guardians were requested to complete the study questionnaire without assistance before commencing the oral examination. Information including sociodemographic characteristics (gender, age, height, weight, parents' education level, and parents' income); disorders or diseases during gestation (e.g., premature birth); physical conditions (systemic diseases and discomfort); dietary habits (including frequency of sweets, iron and zinc consumption); and oral hygiene habits (tooth brushing frequency and oral examination frequency) of the participants were collected using the questionnaires. Parents were also asked to disclose the medical history and any discomfort suffered by their children.

The intraoral examination of children was performed in the morning (9.00–11.00 am) by an experienced dentist, who was qualified to score the caries status of the children according to the decayed, missing, and filled teeth (dmft) and decayed, missing, and filled surfaces (dmfs) indices. According to the American Academy of Pediatric Dentistry (2018) (16), dental caries is defined as the presence of decayed (non-cavitated or cavitated lesions), missing (due to caries), or filled tooth in any primary tooth in a child. The visible plaque index (VPI) was used to assess bacterial plaque accumulation. Using the previously described criteria (11, 12), the buccal and lingual surfaces of all deciduous teeth were examined, and BTS was recorded as black lines or dots firmly adhered to the tooth surface and parallel to the gingival margin. The same examiner performed all the clinical oral examinations. To assess the intra-examiner reliability, we reexamined 10% of the participants and the Cohen's kappa value (κ) was > 0.8 for all parameters.

For further oral microbiome analysis, the following selection criteria were applied: (1) good state of general health; (2) no intake of antibiotics for the last 3 months; (3) mean VPI < 1; (4) dmft and dmfs = 0. Among the children with BTS, 21 children who met the above criteria were included in the BTS group. The control group included 48 BTS free children who matched the age, gender and kindergarten with the BTS group.

### DNA Extraction and Sequencing

The children were asked not to consume any food or drink for at least 2 h prior to sample collection. In the BTS group, we obtained specimens of saliva (BS), supragingival plaque (BP), and BTS plaque (BTSP). In the control group, we collected saliva (S) and supragingival plaque (P) samples.

Supragingival plaque (~1–2 mg) and BTS samples were collected from tooth surfaces using sterile carbon curettes. For the BTS group, the BP and BTSP samples were collected from the non-stained and black-stained tooth surfaces, respectively. All specimens were stored in sterile Eppendorf tubes (5 ml) containing 400 μl of sterile water. Unstimulated whole mouth saliva (2–3 ml) was collected from each participant with a sterile Eppendorf tube (5 ml) using a funnel. All the specimens were immediately transferred to a dry ice-containing bubble chamber and placed in a freezer (−80°C) within 4 h until further experimentation.

The microbial DNA was extracted using the soil DNA Kit (EZNA^®^ Omega Bio-tek, Norcross, GA, USA) and further analyzed for quality with agarose gel (1%) electrophoresis. The purity and final concentration of DNA were evaluated using a UV-vis spectrophotometer NanoDrop 2000 (Thermo Scientific, Wilmington, MA, USA). To amplify the bacterial 16S-rRNA gene (V3-V4 hypervariable segments), we used the primers 338F(5-ACTCCTACGGGAGGCAGCAG-3) and 806R(5-GGACTACHVGGGTWTCTAAT-3) with a thermocycler PCR system (GeneAmp 9700, ABI, USA). The following PCR protocol was applied: denaturation (3 min; 95°C, 27×30 s cycles at 95°C), annealing (30 s; 55°C), and elongation (45 s; 72°C), followed by final extension (10 mins; 72°C) (17). All specimens were run in triplicate using aliquots of 20 μL (4 μL of 5 × FastPfu Buffer, 0.8 μL of each primer (5 μM), 2 μL of deoxynucleoside triphosphate (dNTP; 2.5 mM), DNA template (10 ng), and 0.4 μL of FastPfu polymerase). PCR yields were extracted using agarose gels (2%) and the AxyPrep DNA Gel Extraction Kit (Axygen Biosciences, Union City, CA, USA). Quantification and further purification were conducted utilizing QuantiFluor™-ST (Promega, Fitchburg, WI, USA). After purification, the amplicons in equimolar paired-end sequences (2 × 300) were pooled using the Illumina MiSeq platform (Illumina, San Diego, CA, USA) according to the instructions from Majorbio Bio-Pharm Technology Co. Ltd. (Shanghai, China). The raw sequencing data for this study can be accessed through the National Center for Biotechnology Information Sequence Read Archive (Accession# PRJNA750886).

### Bioinformatics and Statistical Analyses

Trimmomatic and FLASH software (v1.2.11) were used for the interpretation of the sequencing data as previously reported (18, 19). The operational taxonomic units (OTUs, similarity cutoff rate of 97%) were clustered using UPARSE v7.1 software (http://drive5.com/uparse/) (20). The recognition and elimination of chimeric sequences were performed using UCHIME.

Each 16S-rRNA gene sequence was analyzed using the RDP Classifier algorithm (http://rdp.cme.msu.edu/) along with the Human Oral Microbiome Database (HOMD) (v15.2). The online Majorbio I-Sanger Cloud Platform (www.i-sanger.com) was used for further data analysis.

Characteristic sequences were split at various classification levels (from phylum to species). Bacterial richness diversity was assessed with the α index. QIIME (v1.9.1) was used to perform principal coordinate analysis (PCoA) based on the normalized weighted Unifrac distance matrices (21). Differences in the composition of the microbiota were compared through analysis of similarities (ANOSIM) while the relative abundances (mean differences) were assessed at the species level using the linear discriminant analysis (LDA) effect size (LEfSe) (21). A fixed α value (0.05) was applied to conduct the Kruskal–Wallis H and Wilcoxon tests among the groups, controlling the false discovery rate (FDR) for multiple comparisons. The logarithmic LDA scores were analyzed using a threshold of 3.0. The association of microbial community structure with affecting factors was evaluated by variation partitioning along with distance-based redundancy analysis (db-RDA). The phylogenetic investigation of communities by reconstructing unobserved states (PICRUSt) was used to predict the functional profiles of the microbial communities (22). Genome functional annotation was performed on Clusters of Orthologous Groups of proteins (COG) and analyzed by disintegrating the predicted functional composition profiles into level-3 pathways in the Kyoto Encyclopedia of Genes and Genomes (KEGG) database (23).

Data were analyzed using SPSS software (v20, IBM, NY, USA) with the significance level set at 5%. The significance of differences in continuous variables between the BTS and control groups was compared using the Student's *t*-test. Furthermore, we applied the Chi-square test to analyze the differences in clinical and lifestyle factors in both groups. Factors associated with BTS in children were evaluated using binary logistic stepwise regression. All variables that had a *P* value <0.2 in the bivariate analyses were selected for the regression model. The association of the VPI values with the relative abundance of each species and number of black teeth was evaluated by Spearman's correlation analysis.

## Results

### General Characteristics of Participants

In total, 282 children were invited to participate in the investigation. The parents of 271 children completed the questionnaire, with a response rate of 96.1%. Finally, 88.7% (250/282) children received the clinical oral examination.

We analyzed the demographic and clinical data of 250 (130 boys, 120 girls) children. BTS was observed in 12.40% (31/250) of the children, with a mean of 13.7 black-stained teeth. In terms of gender distribution, there was no significant difference between the BTS group and control group (*P* = 0.269). The mean VPI was significantly lower in the BTS group (0.29 ± 0.16) than in the control group (0.47 ± 0.25, *P* = 0.003). The mean values for dmft and dmfs were also significantly lower in the BTS group than in the control group (dmft 1.1 ± 1.9 vs. 1.3 ± 2.4, *P* = 0.037; dmfs 2.1 ± 2.8 vs. 3.1 ± 4.7, *P* = 0.004, respectively). The questionnaire results were shown in Supplementary Table 1. According to the logistic regression analysis, children who consumed marmalade or honey less frequently (*P* = 0.033) and received fluoride application regularly (*P* = 0.007) had a lower likelihood of having BTS (Table 1).

**Table 1:** Application of multiple logistic regression model for black tooth stains.

**Variables**	**Odds ratio**	**95%CI**	***P***
Frequency of marmalade and honey intake			0.033
≥1 × /day	7.23	0.83–62.97	
1–6 × /week	2.41	0.28–20.46	
Seldom/never^a^			
Apply fluoride regularly			0.007
Yes	0.15	0.05–0.50	
No	0.66	0.23–1.89	
Not clear^a^			

a *Reference group*.

*CI, Confidence interval*.

### Sequencing Characteristics

All participants that met the inclusion criteria were selected for 16S rRNA gene sequencing. The two groups were matched in terms of gender and age (*P* > 0.05). The BTS group had significantly lower mean VPI than the control group (*P* = 0.003, Supplementary Table 2).

A total of 159 samples were collected for further analysis. The specimens consisted of five subgroups: BS (21 samples), BP (21 samples), BTSP (21 samples), S (48 samples), and P (48 samples).

**Figure 1:**
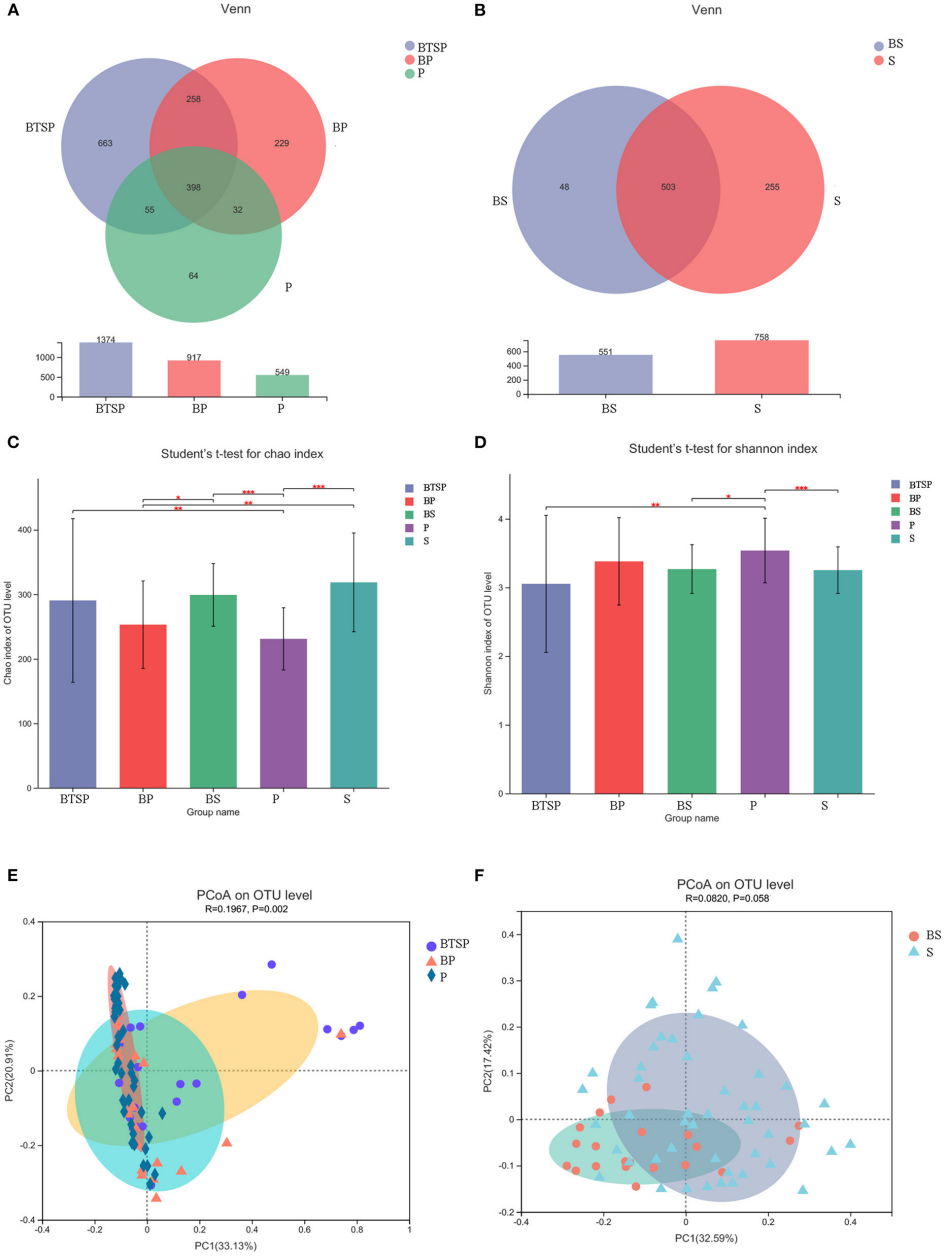
Characteristics and diversity of saliva and plaque microbiota. **(A)** Venn diagram depicting OTU distributions of plaque samples. **(B)** Venn diagram of saliva samples. **(C)** Chao index comparing the α diversity; *P < 0.05, **P < 0.01, ***P < 0.001. **(D)** Shannon index; ^*^P < 0.05, **P < 0.01, ***P < 0.001. **(E)** PCoA showing the differences in the plaque microbiome community structure based on the weighted normalized UniFrac distance with ANOSIM analysis. **(F)** PCoA of the saliva microbiome.

A total of 6,755,214 high-quality reads were generated with a mean of approximately 43,302 reads per specimen. Sequence OTU clustering and notation (at 3% divergence level) identified 14 phyla, 35 classes, 63 orders, 113 families, 221 genera, 452 species, and 1,771 OTUs. Rarefaction revealed the near-complete sampling of saliva, plaque, and the BTS community (Supplementary Figure 1). The shared and unique OTUs in the five subgroups were illustrated in a Venn diagram (Figure 1). In both groups, the majority of OTUs (503) were preserved and shared in saliva. Notably, 398 OTUs were detected in all three plaque subgroups (BTSP, BP, and P), and 663 OTUs were unique in the BTSP subgroup.

MOTHUR was used to identify and quantify bacterial taxa from the phylum level to the species level through taxonomic assignment against the reference HOMD database. The phyla *Proteobacteria, Bacteroidetes, Actinobacteria*, and *Fusobacteria* constituted ~94% of the microbial composition in all five subgroups. The most prevalent genera in the plaque samples were *Neisseria, Leptotrichia, Pseudomonas, Actinomyces, Corynebacterium, Capnocytophaga*, and *Streptococcus. Neisseria, Streptococcus, Veillonella, Prevotella, Haemophilus*, and *Porphyromonas* were the most prevalent genera in the saliva microbiota. Supplementary Figure 2 presents the bacterial average relative abundances at other levels.

### Diversity of Saliva and Plaque Microbiota

The α diversity of the saliva and plaque microbiomes was analyzed using the Chao index (Figure 1C) and Shannon index (Figure 1D). The Chao index showed that the BTSP samples had a higher OTU richness than the P samples (BTSP vs. P, *P* = 0.006). Comparing the Shannon index data indicated that the P samples had significantly greater microbial diversity than the BTSP samples (BTSP vs. P, *P* = 0.007). The control group had a more balanced environment than the BTS group. The saliva samples from the two groups showed no statistical differences in terms of diversity (Chao *P* = 0.288, Shannon *P* = 0.861, respectively). Both the Shannon and Chao indices also showed no significant differences in the supragingival plaque samples (BP vs. P, Chao *P* = 0.774, Shannon *P* = 0.356, respectively).

Upon analyzing the similarities and differences in the overall microbial composition and structure (PCoA and ANOSIM), insignificant variations were detected in the structure of plaque microbiota among the BTSP, BP, and P samples (Figure 1E; *P* = 0.002, R = 0.197). In addition, the bacterial community composition of the saliva samples showed no significant difference between the BTS and control groups (Figure 1F; *P* = 0.058, R = 0.082).

### Characterization of Saliva and Plaque Microbiota Associated With Black Tooth Stain

**Figure 2:**
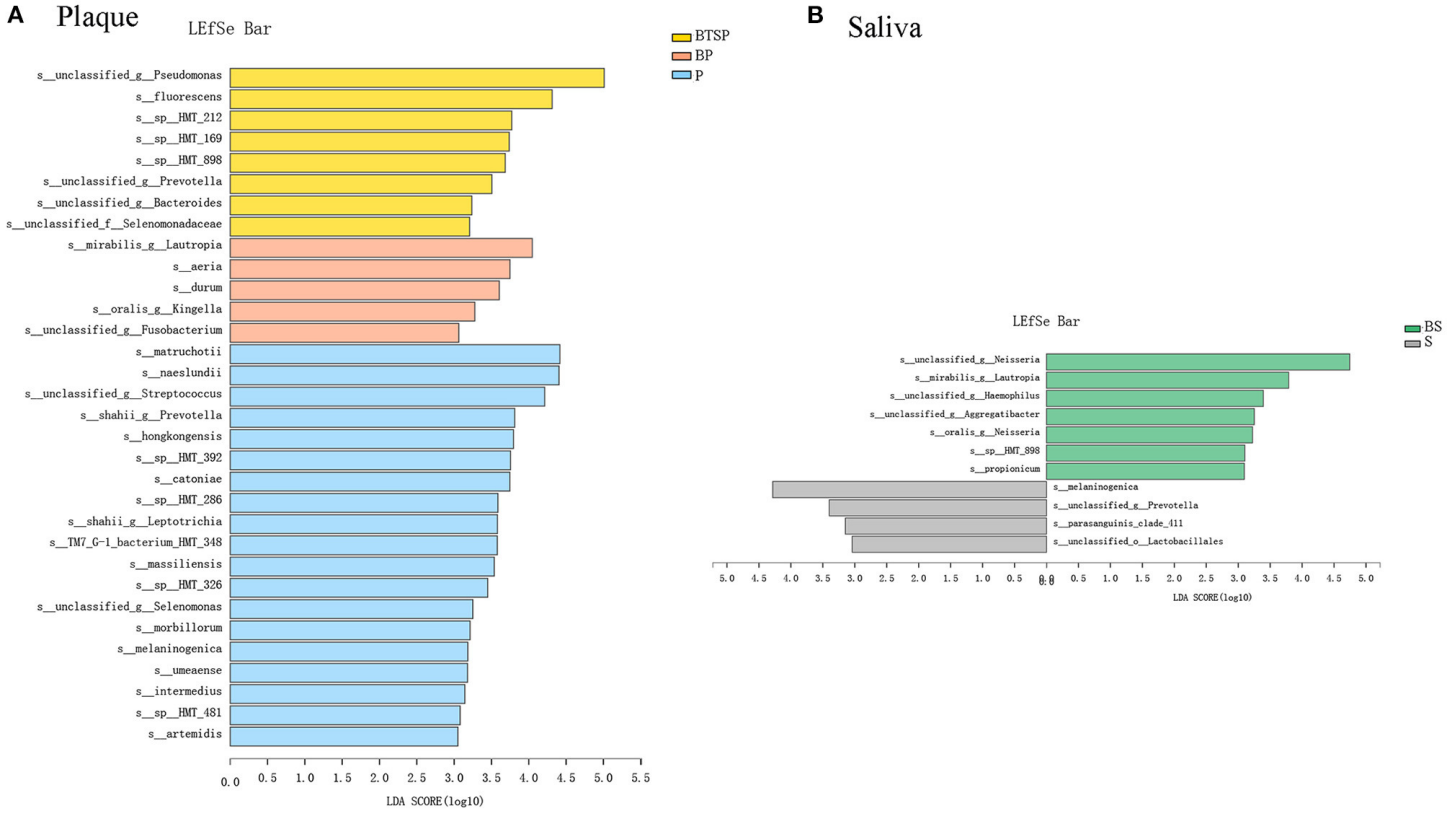
Comparisons of the relative bacterial abundance in the BTS and control groups; LDA revealed significant variations in the levels of bacterial species [LDA Score (log10) > 3]. **(A)** Plaque samples. **(B)** Saliva samples.

To classify microbiota associated with BTS in saliva and plaque samples, the relative bacterial abundances among the groups were compared using LEfSe analysis and the LDA scores at the species level (Figure 2, LDA score (log10) > 3). The results revealed various BTS-enriched species. We distinguished the BTS microbiome based on eight biomarkers, including *Pseudomonas fluorescens, Leptotrichia sp._HMT_212, Actinomyces sp._HMT_169*, and *Aggregatibacter sp._HMT_898*. Five bacterial species, including *Lautropia mirabilis, Rothia aeria*, and *Corynebacterium durum*, were more abundant in the BP samples. P samples from the control group were characterized by 19 bacterial species, including *Corynebacterium matruchotii, Actinomyces naeslundii*, and an unclassified *Streptococcus* species (Figure 2A). We also applied the Kruskal-Wallis H test for further comparison of the relative abundance of species in the plaque microbiota based on the top 30 abundant species in the three plaque subgroups (Supplementary Figure 3A).

Compared to the S samples from the control group (Figure 2B), the relative proportions of seven species from the genera *Neisseria, Lautropia, Haemophilus*, and *Aggregatibacter* were higher in the BS samples. The distributions of four species, including *Prevotella melaninogenica* and *Streptococcus parasanguinis clade 411*, were highly abundant in the control group. The Wilcoxon rank-sum test results for the 30 most abundant species in the two saliva groups are shown in Supplementary Figure 3B.

### Correlation Between Plaque and Saliva Microbiota and the Number of Teeth With Black Tooth Stains

**Figure 3:**
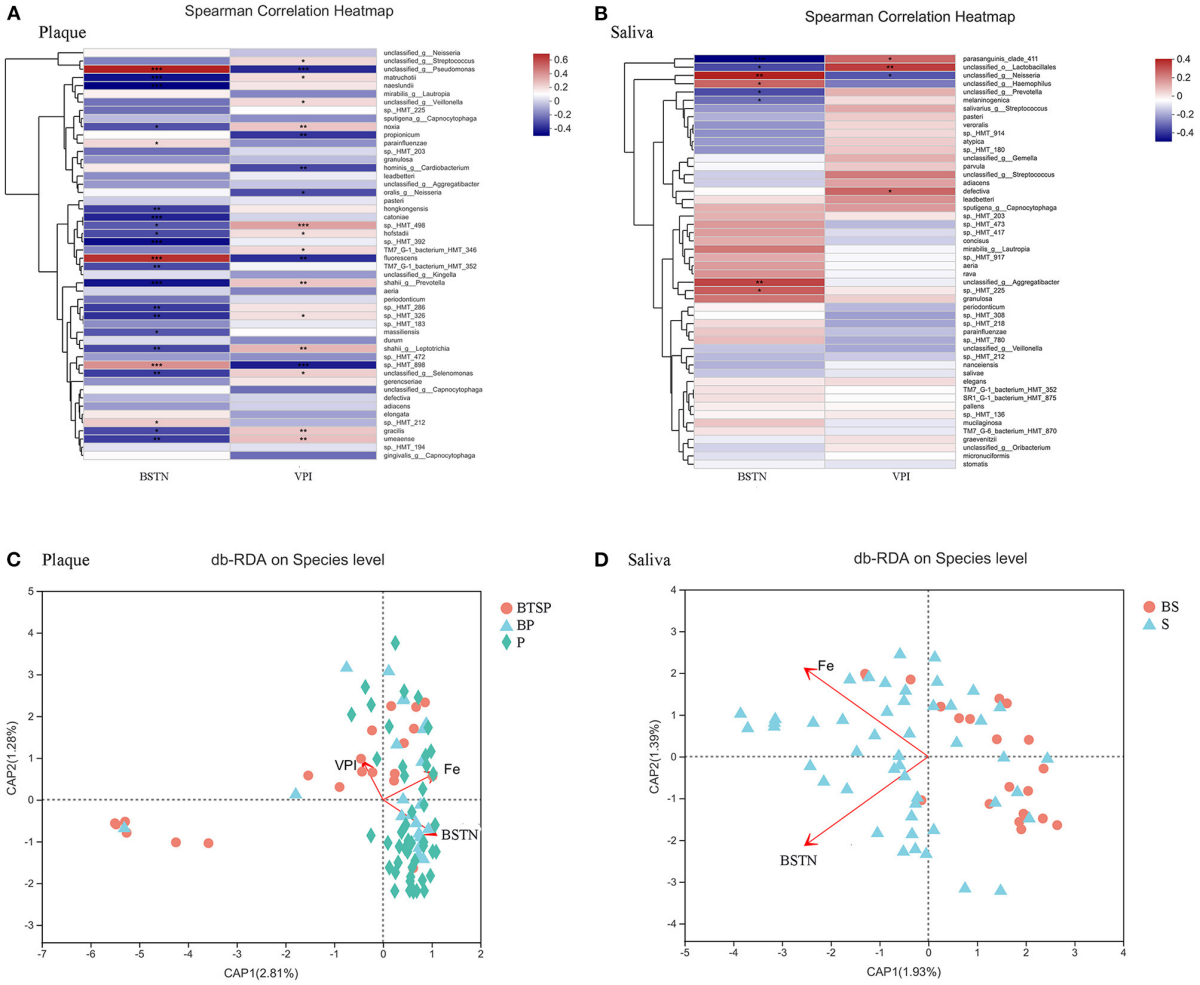
Factors related to the bacterial community structure. BSTN: black-stained teeth number; VPI: visible plaque index; Fe: iron supplementation period; *P < 0.05, **P < 0.01, ***P < 0.001. **(A)** Spearman's correlation analyses for relative abundance (%) of the 50 most abundant species and related environmental factors in plaque samples. **(B)** Spearman's correlation analyses of saliva samples. **(C)** db-RDA plot at the species level in plaque samples. **(D)** db-RDA plot of saliva samples.

To identify the association of plaque and salivary microbiota with the number of teeth with BTS, further correlation analyses of the relative abundance (%) was conducted with Spearman's correlation using the top 50 species in the plaque and saliva microbiota and the black-stained teeth number (BSTN) (Figure 3). Specific bacterial species related to VPI were also analyzed. In the plaque samples, five bacterial species were positively correlated with the BSTN, while 17 bacterial species were negatively correlated (Figure 3A). The relative abundances of *Corynebacterium matruchotii* (*r* = −0.397, *P* < 0.001)*, Actinomyces naeslundii* (*r* = −0.438, *P* < 0.001), and *Leptotrichia HMT_392* (*r* = −0.406, *P* < 0.001) showed a decreasing trend with the increase of BSTN. *Pseudomonas fluorescens* (*r* = 0.658, P < 0.001), an unclassified species from *Pseudomonas* (r = 0.696, *P* < 0.001), and *Aggregatibacter sp._HMT_898* (*r* = 0.411, *P* < 0.001) were positively associated with the BSTN. Negative correlations to the VPI values were found in the relative abundances of *Pseudomonas fluorescens* (*r* = −0.332, *P* = 0.001), an unclassified species from *Pseudomonas* (*r* = −0.342, *P* < 0.001), and *Aggregatibacter sp._HMT_898* (*r* = −0.373, *P* < 0.001). Noticeably, most species, such as *Prevotella shahii, Leptotrichia shahii*, and *Lachnoanaerobaculum umeaense*, showed opposite correlation relationships with BSTN and VPI.

In saliva, eight bacterial species were significantly correlated with the BSTN (Figure 3B). For instance, *Streptococcus parasanguinis_clade_411* (*r* = −0.421, *P* = 0.0003) had a significantly negative correlation with the BSTN. Moreover, a noticeable positive association of *Streptococcus parasanguinis_clade_411* with VPI values (*r* = 0.256, *P* = 0.034) was observed.

### Environment Factors Associated With Plaque and Saliva Bacterial Community

To explore whether other environment factors had any additional influence on the microbiota, the collinearity among various environmental factors (clinical and questionnaire data) was assessed by measuring the variance inflation factor (VIF). The factors with VIF > 10 were excluded from the subsequent analysis. The db-RDA based on the weighted normalized Unifrac distance matrix was determined to explore whether other environment factors had any additional influence on the saliva and plaque microbiota communities and composition. The species level db-RDA results showed a relationship between the iron supplementation period and the plaque (Figure 3C, *r*^2^ = 0.15, *P* = 0.002) as well as the saliva (Figure 3D, *r*^2^ = 0.14, *P* = 0.006) microbial community structures in these children. The plot indicated a strong inverse connection between the plaque microbiota and the values of VPI (*r*^2^ = 0.10, *P* = 0.012) and BSTN (Figure 3C, *r*^2^ = 0.20, *P* = 0.001). In addition, a correlation was also found between the saliva microbiota and BSTN (Figure 3D, *r*^2^ = 0.27, *P* = 0.001).

### Functional Features of the Microbiota Correlated With Black Tooth Stain

**Figure 4:**
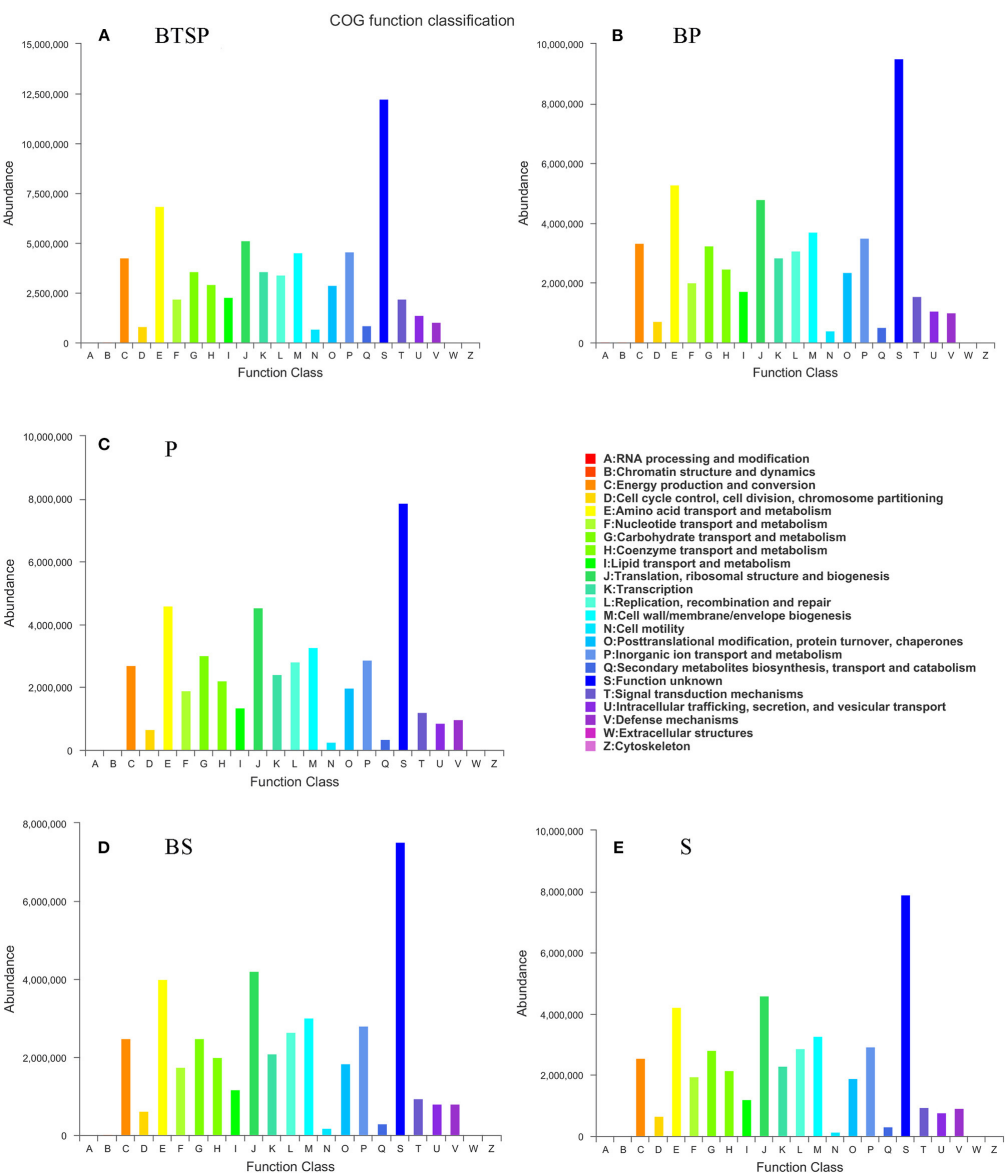
Functional annotation of the microbiome based on Clusters of Orthologous groups of proteins (COG). **(A–E)** BTS plaque from BTS group (BTSP), supragingival plaque from BTS group (BP), supragingival plaque from control group (P), saliva from BTS group (BS) and saliva from control group (S), respectively.

To analyze the functional alterations in the microbiota, PICRUSt was performed to determine the microbial functional composition profiles. The COG database indicated that amino acid transport and metabolism, translation, ribosomal structure and biogenesis, and cell wall/membrane/envelope biogenesis were enriched in all five subgroups (Figure 4; Supplementary Figure 4).

**Figure 5:**
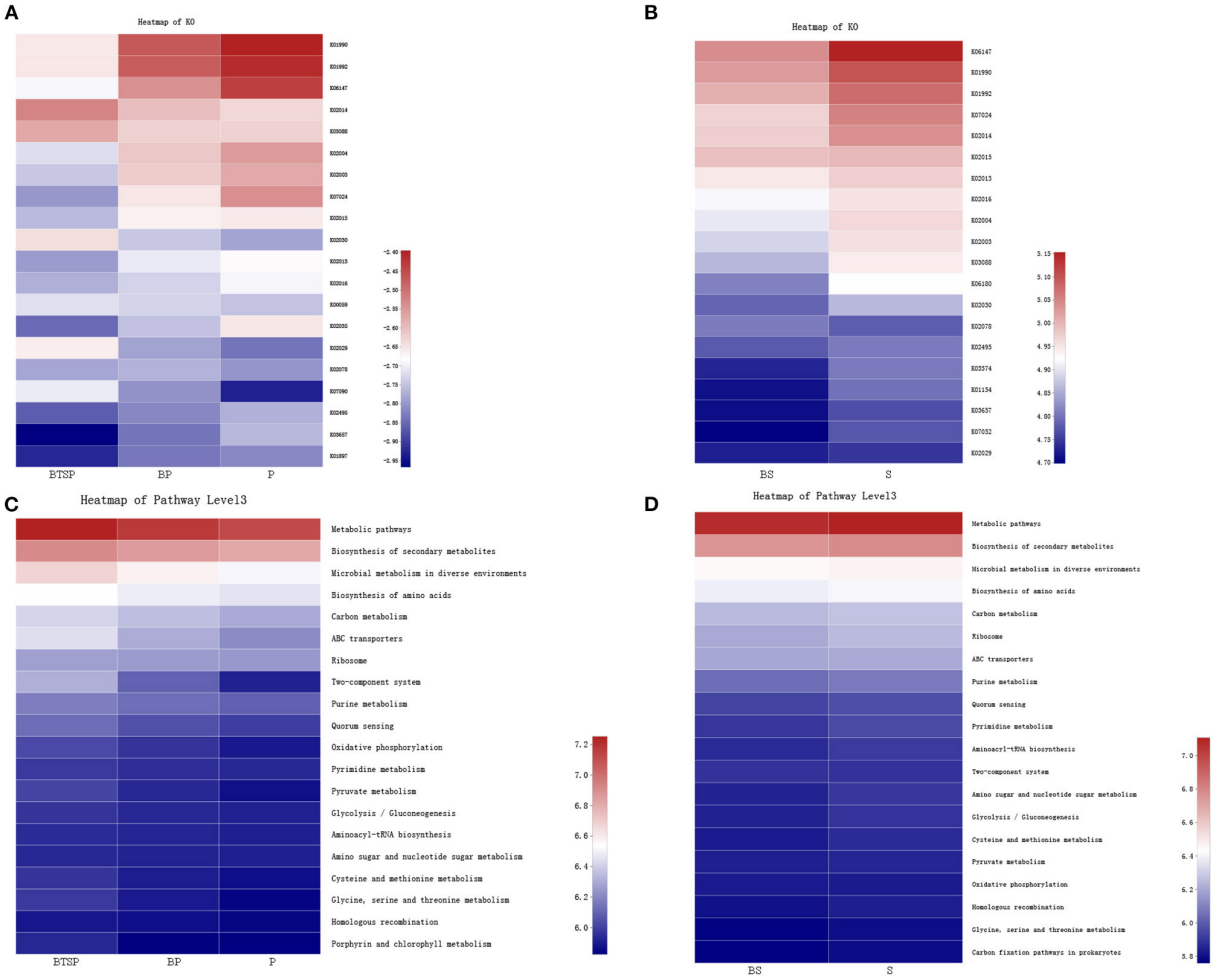
Metabolism alterations in the microbiome. The color in the heatmap represents the relative abundance according to the right panel: **(A)** Predicted functional profile with KEGG Orthology (KO) in plaque samples. **(B)** KO of saliva samples. **(C)** KEGG pathways (level 3) in plaque samples. **(D)** KEGG pathways (level 3) in saliva samples.

**Figure 6:**
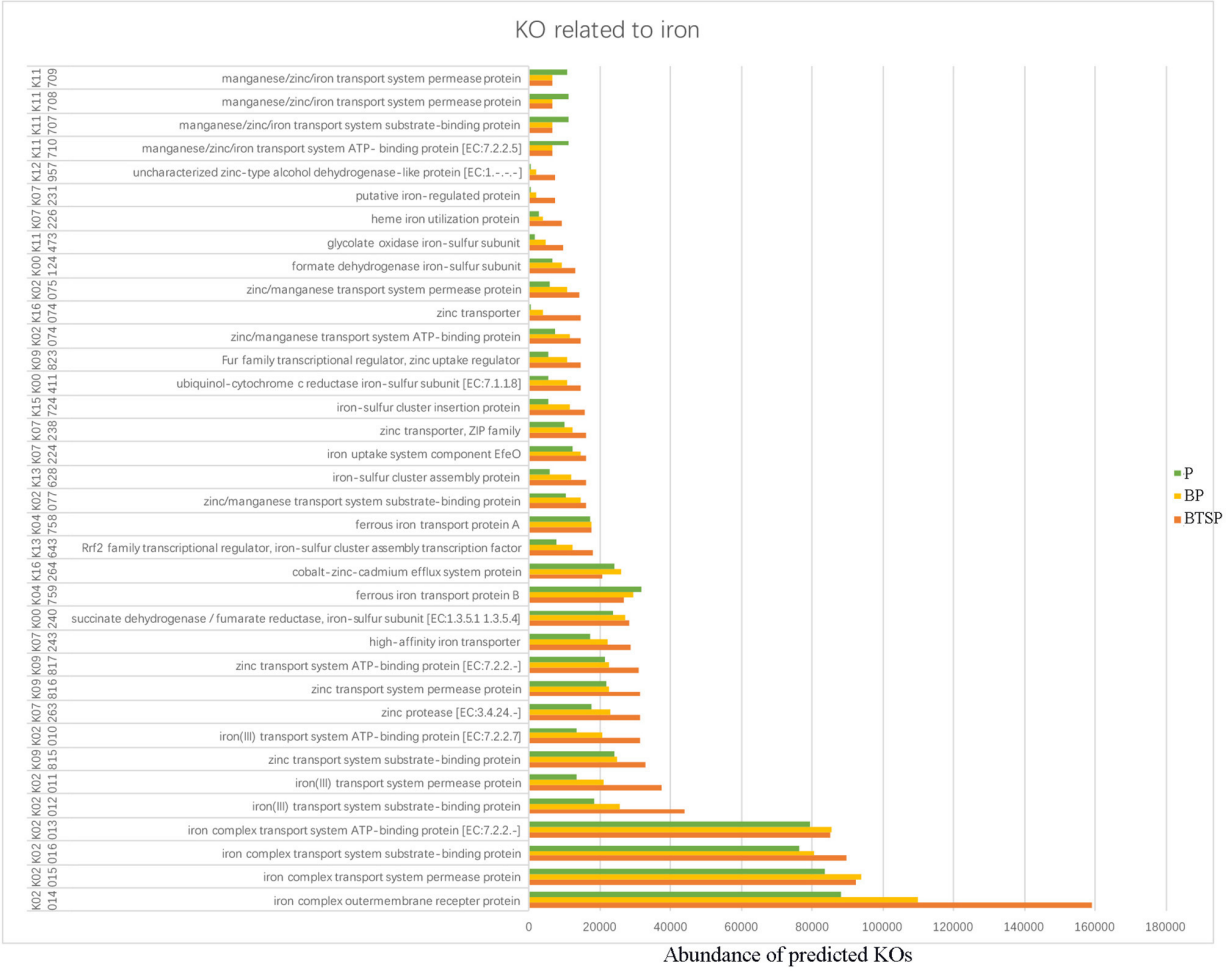
The KEGG Orthology (KO) groups related to iron in plaque samples.

Using the KEGG database, we obtained the KEGG Orthology (KO) and correlated the microbial functional features with important proteins found in the saliva and plaque samples. The 20 most abundant KO groups were visualized with heatmaps (Figure 5). Notably, in the saliva microbiota, iron complex transport system-related proteins (K02013-K02016) were more enriched in the control group (Figure 5B). In the plaque samples, these proteins were more enriched in the BTSP microbiota, especially the iron complex outer membrane receptor protein (Figure 5A), showing an opposite tendency compared to the saliva samples. In addition, proteins such as the ABC-2 type transport system permease protein, ABC-2 type transport system ATP-binding protein, sucrose-6-phosphatase [EC:3.1.3.24], and ATP-binding cassette, exhibited increased levels in the control group saliva and plaque specimens. To further evaluate the function of iron in the bacteria in the BTSP specimens, KO groups associated with iron were selected (Figure 6). It was found that the KO groups enriched in the BTSP samples contained ferric iron (K02010, K02011, K02012). Moreover, in KEGG pathways for the plaque samples (level 3), pathways related to glycolysis/gluconeogenesis, cysteine and methionine metabolism, pyruvate metabolism, serine, glycine, and threonine metabolism were more abundant in the BP group (Figure 5C).

## Discussion

The present study assessed the prevalence and epidemiological features related to BTS among preschool children in China. For this purpose, we collected data related to the sociodemographic, lifestyle, and oral hygiene habits of the children using an objectively structured questionnaire. Microbial saliva and plaque specimens from the participants were analyzed to determine the microbial features correlated with BTS.

Since the incidence of oral diseases (such as caries and periodontitis) is relatively low in junior kindergarten children, and the microbial basis of BTS in the oral environment remains largely unknown, first-year kindergarten children (3–4 years) are considered good candidates for studying BTS and extracting useful scientific data. In this study, BTS was observed clinically in 12.4% of 250 preschool children. The majority of children in the BTS group had deposition of black stains on more than eight teeth. The percentage of BTS in this study was similar to that reported in other studies (11, 13) and higher than the prevalence (9.9%) reported in our previous study (24). The dental caries indicators (dmft and dmfs indices) were significantly lower in the BTS group in this study. These finding are in agreement with our previous study (24). Children with BTS on permanent dentition tend to have a lower prevalence of caries (2, 10, 25–27). In this study, the mean VPI was also remarkably lower in children with BTS compared to the value in the control group, which may be responsible for the lower incidence of dental caries. Moreover, bacterial species (such as *Aggregatibacter sp._HMT_898*) showed an inverse relationship with the BSTN and VPI values. The db-RDA plot also indicated a strong inverse relationship between the plaque microbiota and the values of VPI and BSTN. The improved oral health of the participants with BTS may be attributed to more attention in maintaining the oral hygiene to relieve or control the expansion of BTS.

Analysis of related sociodemographic factors and oral health habits revealed a lower risk of BTS in children with less frequent intake of marmalade and honey and regular fluoride application. These findings provide evidence of the influence of the oral hygiene behavior and dietary habits on BTS in children. In the present study, we observed a preventive role of BTS against dental caries. In addition, diet and consumption frequency may alter the composition of dental plaque microbiota (28). These results suggest that the presence of BTS may limit the progression of caries (10). Therefore, reducing the consumption frequency of cariogenic foods such as marmalade and honey may reduce the growth of cariogenic bacteria, thereby encouraging a favorable environment for bacteria associated with the development of BTS. Fluoride application on a regular basis may facilitate a higher pH in the oral cavity, which may be favorable for the deposition of BTS. However, these hypotheses require validation and further investigations.

BTS is a distinct form of dental plaque that is deposited by oral microorganisms (29). Since some of these bacteria are also related to various oral diseases, we selected children with good oral condition (without caries) for further oral microbiome analysis. BTS is deposited mainly on the lingual surfaces of mandibular incisors that are directly exposed to saliva secreted by the submandibular glands. Considering the vital role of saliva in BTS, we investigated plaque and saliva samples for further microbial features analysis. The collection of bacterial plaque using metallic instruments may contaminate the specimens with metallic ions (30). Thus, in this study, we used graphite curettes to collect the BTS samples.

In this study, α and β diversity indices indicated no significant differences in the microbial structure of saliva in children with unstained teeth and those with BTS. However, comparisons of the Chao and Shannon indexes in plaque samples revealed that BTSP samples had a higher OTU richness but a lower evenness than the control P samples, indicating the control group had a more balanced environment than the BTS group. ANOSIM analyses of the plaque microbiota also indicated significant differences in β diversity in the three plaque groups. Han et al. analyzed the salivary microbiome diversity and reported significant differences in the microbial diversity of the BTS and control groups (7). Chen et al. performed 16S rRNA sequencing and reported no statistically significant differences in the diversity and Unweighted Unifrac PCoA in the supragingival plaque microbiome between children with (*n* = 10) and without (*n* = 10) BTS (31). In contrast, Sheth et al. compared the microbiomes of BTS and white plaque in adults and reported poorer species diversity in the BTS microbiome than in the white-plaque microbiome (32). The differences in diversity trends may be attributed to variations in the ages, dietary habits and life habits of the volunteers and the tendency of dysbiosis to develop over time.

The BTS plaque microbiome was characterized by eight microbial biomarkers. *Actinomyces* has been reported to have the capability to produce black or brown pigmentation (33). The predominance of *Actinomyces* in the oral cavity is associated with BTS in children (4, 8, 31). Furthermore, hydrogen sulfide produced by oral microorganisms (such as *Actinomycetes*) can react with ferric ions present in saliva, resulting in the formation of BTS. The adhesion of *Actinomyces naeslundii* may also reduce the rate of caries formation (34, 35). Weltzien et al. reported a significant increase in *Actinomyces naeslundii*, which reduced caries progression in BTS children (11). Similarly, *Actinomyces* colonization enhances the level of *Actinomyces* antibodies, which also have an inhibitory effect on caries (35). Interestingly, we found a high prevalence of *Actinomyces naeslundii* in supragingival plaque from the control group, whereas *Actinomyces sp._HMT_169* was more prevalent in the BTS plaque. There are several reasons for these differences in the results. First, *Actinomyces* reported in some previous studies were analyzed at the genus level or in saliva samples (15, 26, 32) and specific *Actinomyces* species were not defined in BTS plaques. Second, the participants in our study were all caries-free and had low VPI levels. The *Actinomyces* abundance related to caries may not be obvious in this study and the function of specific *Actinomyces* species in BTS formation and the associated pathophysiology remain unclear. Moreover, significantly higher numbers of *Rothia aeria* were found in black-stained plaques in the present study. *Rothia* belongs to the *Actinomycetaceae* family, which are filamentous, gram-positive, facultative anaerobic cocci and present many features comparable to those of *Actinomyces* (26).

In the present study, *Aggregatibacter sp._HMT_898* was highly abundant in BP. When comparing the saliva samples, the relative proportions of species from *Aggregatibacter* were higher in the BTS group than in the control group. Similarly, Celik et al. also reported a significantly higher count of *Aggregatibacter* in black-stained plaques (26). *Aggregatibacter actinomycetemcomitans* is a Gram-negative periodontal pathogen that may produce black pigment and plays a role in the formation of BTS. The prevalence of *Aggregatibacter actinomycetemcomitans* was reported to be significantly higher in BTS plaque samples (70%) than in black stain-free plaque samples (20%) (8). In contrast, real-time PCR analysis revealed no significant differences in the prevalence of *Aggregatibacter actinomycetemcomitans* in teeth with and without BTS (11). In the present study, no significant difference was detected in the prevalence of *Aggregatibacter actinomycetemcomitans* while comparing the plaque from teeth with and without BTS. These different results indicated associations between complex microbial interactions and BTS formation in the oral cavity, and their roles need to be investigated further.

According to microbiota functions analysis, pathways related to glycolysis/gluconeogenesis, cysteine and methionine metabolism, pyruvate metabolism, serine, glycine, and threonine metabolism were highly abundant in the BP group. These findings are similar to those of previous studies (26, 36), suggesting the existence of a hyperactive metabolic state in teeth surfaces with BTS plaques.

Various nutritional substance present in the dental plaque may fortify bacteria to produce metabolic products that may facilitate the formation of BTS; for example, consuming iron-rich diets or supplements has been reported to increase the deposition of BTS by increasing the availability of iron in the oral fluids (37). The species level db-RDA results in this study indicated a relationship between the iron supplementation period and the plaque and saliva microbial community structures in these children. However, we found no association between the iron supplementation period and BTS prevalence in the questionnaire data. Similarly, Prskalo et al. (38) and Veses et al. (25) reported no correlation of iron supplements with BTS in their investigations. Chen et al. proposed two hypotheses regarding the role of iron in BTS (39). First, BTS is related to bacterial metabolism. The plaque bacteria produce iron compounds, resulting in the formation of black deposits. Secondly, certain factors (for example, diet) may alter the plaque microbiome, thereby encouraging black sedimentation through altered microbial interactions. In the diet survey performed in this study, we found no association between iron or zinc supplements and BTS. However, diet may not be the only factor that affects the formation of BTS (39).

We found that KO groups related to ferric iron (K02010, K02011, K02012) were enriched in BTSP specimens (Figure 6). Moreover, the iron complex transport system and the iron (III) transport system exhibited enhanced activity in the BTS group, while the manganese/zinc/iron transport system showed increased activity in the control group. In addition, the nutrition source and chemical nature of iron may influence biofilm formation (39). The differences in the iron-related KO groups also suggested an association of iron and microbiota with the formation of BTS. However, the mechanism underlying the formation of BTS is still need to be further investigated

Interestingly, in saliva, KO groups related to ferric iron, the iron complex transport system, and the iron (III) transport system were similar or more abundant in the control group. Considering the similar overall microbial composition and structure in the BS and S groups, we propose that the plaque samples may be more representative of BTS-related oral microflora.

There are certain limitations in this study. We did not classify these samples by black tooth stain degree, which might be related to the microbiome composition. Besides, we did not analyze the iron contents in BTS plaque and saliva in our participants. The correlations between plaque and saliva microbiota and the concentrations of iron were not explored in this study. There are several theories about the role of iron in BTS. Reid et al. suggested that the black pigmentation is caused by the formation of ferric sulfide as a result of chemical reactions between the iron present in the saliva or gingival exudate and hydrogen sulfide produced by certain bacteria (40). Another study indicated that the stains contain iron and copper in trace amounts along with a substantial proportion of organic matter (41). The formation of a sulfur and metal ion complex was considered responsible for pigmentation corresponding to zones of high concentrations of sulfur and iron/copper (7). Whether the presence of iron or copper is associated with BTS in primary dentition is still unclear and requires further investigations.

The present study lies in the comprehensive investigation of the microbial composition of BTS. The oral microbiome is considered the main etiological factor in the formation of BTS (5, 11, 15, 26, 31, 32, 36). Although the present study does not provide a definite conclusion regarding the relationship of BTS with oral microbiota, it provides evidence suggesting the involvement of the bacteria metabolic pathway in BTS formation in primary dentition. In addition, the variations detected in the oral microbiota in BTSP samples suggest the involvement of several microorganisms including *Actinomyces*, which contribute to the deposition of BTS. Further studies are required to confirm the dysregulation of metabolic pathways and identify the microbial species responsible for BTS formation.

## Conclusions

The present study explored the epidemiologic and microbial characteristics of saliva and plaque in preschool children with BTS and their counterparts with healthy teeth. Participants with BTS had better oral hygiene and a lower prevalence of dental caries. The likelihood of developing BTS was lower in children with less frequent intake of marmalade and honey and regular fluoride application. The healthy group had a more balanced microbial environment than the BTS group. Microbiome analysis revealed various microbial biomarkers, such as *Pseudomonas fluorescens, Leptotrichia sp._HMT_212, Actinomyces sp._HMT_169*, and *Aggregatibacter sp._HMT_898* in plaques from the BTS group. This information can be used as a basis for understanding the involvement of specific bacteria in the formation of BTS.

## Data Availability Statement

The datasets presented in this study can be found in online repositories. The names of the repository/repositories and accession number(s) can be found in the article/Supplementary Material.

## Ethics Statement

The study was performed with Ethical Approval from the Institutional Ethical Committee of the Ninth People's Hospital, School of Medicine, Shanghai Jiao Tong University, Shanghai, China (Ref No. 2015135). Written informed consent to participate in this study was provided by the participants' legal guardian/next of kin.

## Author Contributions

YZ and XC contributed to the study design, sample collection, clinical examination, data analysis, statistics, interpretation, and drafting and critically revising the manuscript. YZ contributed to data analysis, statistics, and drafting and critically revising the manuscript. G-ZC, RY, and J-YZ contributed to sample collection and drafting and critically revising the manuscript. X-PF contributed to the study design and drafting and critically revising the manuscript. All authors gave final approval and agree to be accountable for all aspects of the work.

## Funding

The study was funded by the National Natural Science Foundation of China (No. 81800967) and Shanghai Municipal Health Commission (Nos. 2019SY027 and 2020YJZX0114).

## Conflict of Interest

The authors declare that the research was conducted in the absence of any commercial or financial relationships that could be construed as a potential conflict of interest.

## Publisher's Note

All claims expressed in this article are solely those of the authors and do not necessarily represent those of their affiliated organizations, or those of the publisher, the editors and the reviewers. Any product that may be evaluated in this article, or claim that may be made by its manufacturer, is not guaranteed or endorsed by the publisher.
