# Supplementary material for: Epidemiological and Microbiome Characterization of Black Tooth Stain in Preschool Children
Source: Front Pediatr. 2022 Jan 26;10:751361. doi: 10.3389/fped.2022.751361 (PMC8826690; doi:10.3389/fped.2022.751361)
Supplement: Supplementary file 1 [file Data_Sheet_1.docx]

**Supplementary material**

**Dynamic alterations of oral microbiota related to halitosis in preschool children**

**Yu Zhang, Rui Yu, Jing-Yu Zhan, Gui-Zhi Cao, Xi-Ping Feng, Xi Chen**

**Table S1.** Relationships between black tooth stains and health behaviors

|  | | N | | % with black tooth stains | | *p* ^a^ | |
| --- | --- | --- | --- | --- | --- | --- | --- |
| Fruit intake† | |  | |  | | 0.634 | |
| ≥1×/day | | 180 | | 12.2 | |  | |
| 1–6×/week | | 63 | | 14.3 | |  | |
| Seldom/never | | 5 | | 0.0 | |  | |
| Dessert intake† | |  | |  | | 0.931 | |
| ≥1×/day | | 58 | | 12.1 | |  | |
| 1-6×/week | | 151 | | 13.2 | |  | |
| Seldom/never | | 36 | | 11.1 | |  | |
| Beverage intake† | |  | |  | | 0.336 | |
| ≥1×/day | | 5 | | 0.0 | |  | |
| 1–6×/week | | 69 | | 8.7 | |  | |
| Seldom/never | | 174 | | 14.4 | |  | |
| Marmalade and honey intake† | |  | |  | | 0.016 | |
| ≥1×/day | | 3 | | 66.7 | |  | |
| 1–6×/week | | 23 | | 8.7 | |  | |
| Seldom/never | | 218 | | 12.4 | |  | |
| Candy intake† | |  | |  | | 0.116 | |
| ≥1×/day | | 28 | | 3.6 | |  | |
| 1-6×/week | | 125 | | 11.2 | |  | |
| Seldom/never | | 91 | | 17.6 | |  | |
| Snack intake before sleeping† | |  | |  | | 0.047 | |
| ≥1×/day | | 93 | | 8.6 | |  | |
| 1-6×/week | | 102 | | 11.8 | |  | |
| Seldom/never | | 64 | | 21.9 | |  | |
| Viscera intake† | |  | |  | | 0.697 | |
| ≥1×/day | | 0 | | – | |  | |
| 1–6×/week | | 29 | | 10.3 | |  | |
| Seldom/never | | 217 | | 12.9 | |  | |
| Food with soy sauce† | |  | |  | | 0.401 | |
| ≥1×/day | | 24 | | 12.5 | |  | |
| 1–6×/week | | 132 | | 15.2 | |  | |
| Seldom/never | | 89 | | 9.0 | |  | |
| Calcium products† | |  | |  | | 0.111 | |
| ≥1×/day | | 42 | | 4.8 | |  | |
| 1–6×/week | | 47 | | 19.1 | |  | |
| Seldom/never | | 146 | | 11.6 | |  | |
| Vitamin D† | |  | |  | | 0.254 | |
| ≥1×/day | | 41 | | 17.1 | |  | |
| 1–6×/week | | 42 | | 14.3 | |  | |
| Seldom/never | | 149 | | 8.7 | |  | |
| Dyspepsia | |  | |  | | 0.665 | |
| Always | | 5 | | 20.0 | |  | |
| Sometimes | | 92 | | 13.0 | |  | |
| Seldom/never | | 144 | | 12.5 | |  | |
| Not clear | | 9 | | 0.0 | |  | |
| Toothbrushing frequency† | |  | |  | | 0.049 | |
| ≥2×/day | | 125 | | 14.4 | |  | |
| 1×/day | | 87 | | 14.9 | |  | |
| Not everyday | | 36 | | 0 | |  | |
| Salt† | |  | |  | | 0.266 | |
| With iodine | | 144 | | 13.9 | |  | |
| Without iodine | | 73 | | 13.7 | |  | |
| Not clear | | 30 | | 3.3 | |  | |
| Iron supplementation | |  | |  | | 0.198 | |
| ≥2 months | | 20 | | 0.0 | |  | |
| <2 months | | 39 | | 15.4 | |  | |
| Seldom/never | | 191 | | 13.1 | |  | |
| Zinc supplementation | |  | |  | | 0.184 | |
| ≥2 months | | 56 | | 5.4 | |  | |
| <2 months | | 81 | | 13.6 | |  | |
| Seldom/never | | 113 | | 15.0 | |  | |
| Fluoride toothpaste use† | |  | |  | | 0.296 | |
| Always | | 68 | | 14.7 | |  | |
| Sometimes | | 50 | | 8.0 | |  | |
| Seldom/never | | 83 | | 16.9 | |  | |
| Not clear | | 42 | | 7.1 | |  | |
| Apply fluoride regularly† | |  | |  | | 0.006 | |
| Yes | | 124 | | 6.5 | |  | |
| No | | 55 | | 16.4 | |  | |
| Not clear | | 63 | | 22.2 | |  | |

^a^ Obtained with the Chi-squared test.

† These variables have some missing data.

N: Number of children examined

**Table S2.** Comparisons of demographic and clinical characteristics between the black tooth stain and control groups

|  | Black tooth stain  (mean ± SD) | Control  (mean ± SD) | | *P* |
| --- | --- | --- | --- | --- |
| Gender (N, % with black tooth stain) |  |  | 0.994 ^a^ | |
| Male | 31 | 32.3 |  | |
| Female | 38 | 28.9 |  | |
| Black-stained teeth number | 15.71±5.68 | 0.00±0.00 | <0.001 ^b^ | |
| Plaque index | 0.20±0.12 | 0.39± 0.22 | 0.003 ^b^ | |

^a^ Obtained from Chi-squared test

^b^ Obtained from Student’s t-test (two groups).

SD: standard deviation; N: Number of children examined


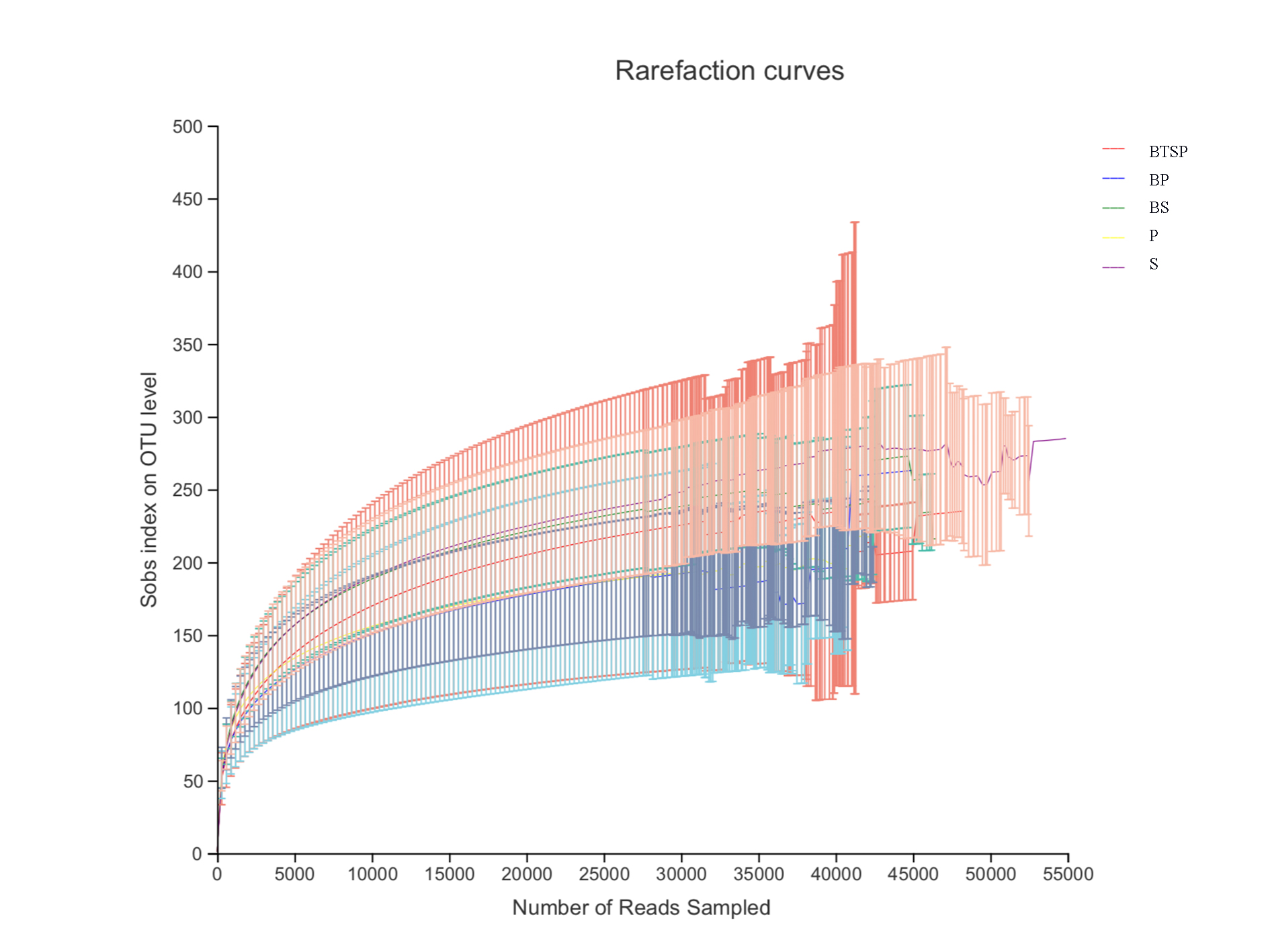


**Figure S1** The rarefaction curves of five subgroups determined by the Sobs index that were generated to evaluate the sequencing depth.

**
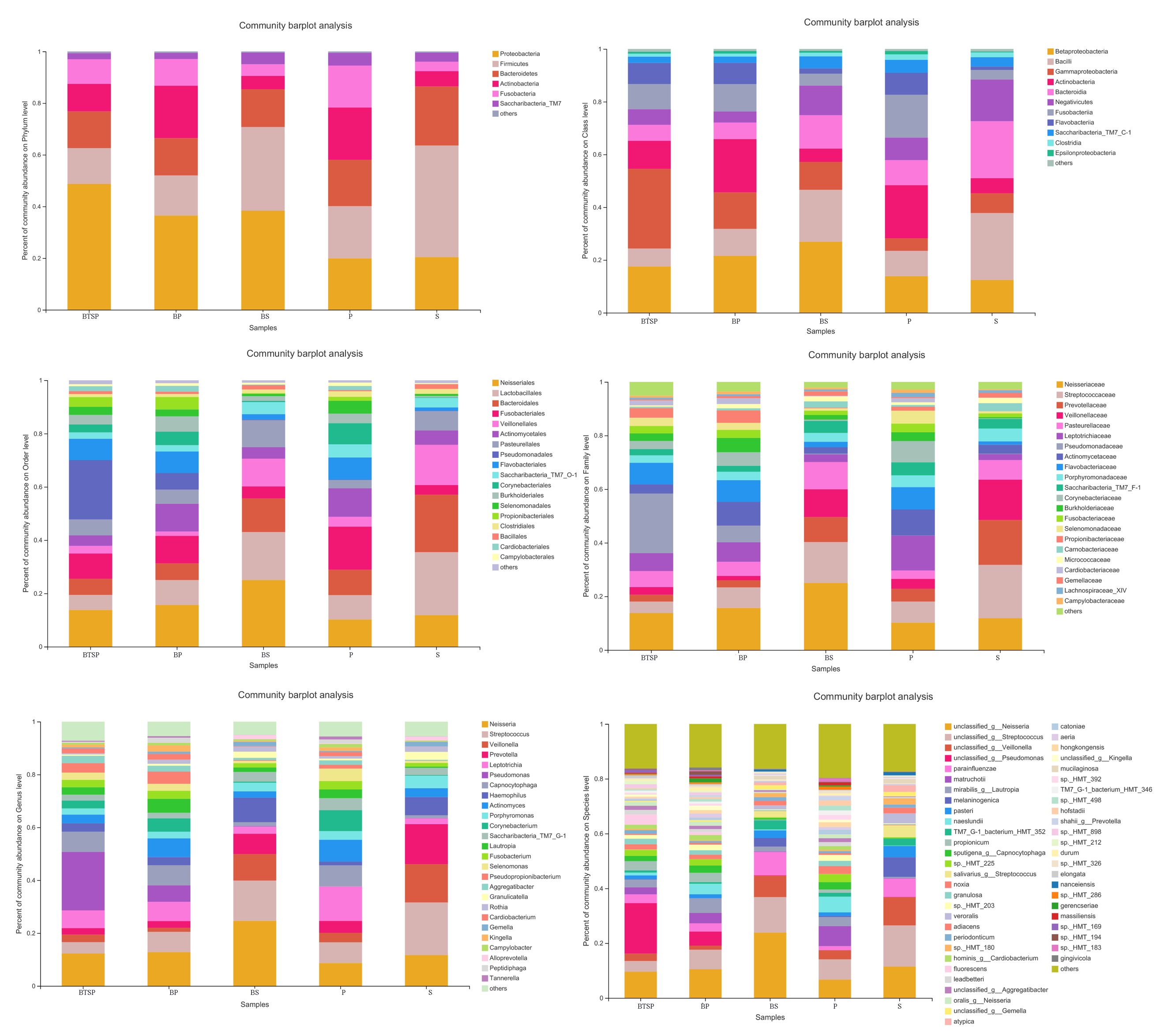
**

**Figure S2.** Composition of bacterial communities in five subgroups from phylum to species levels.


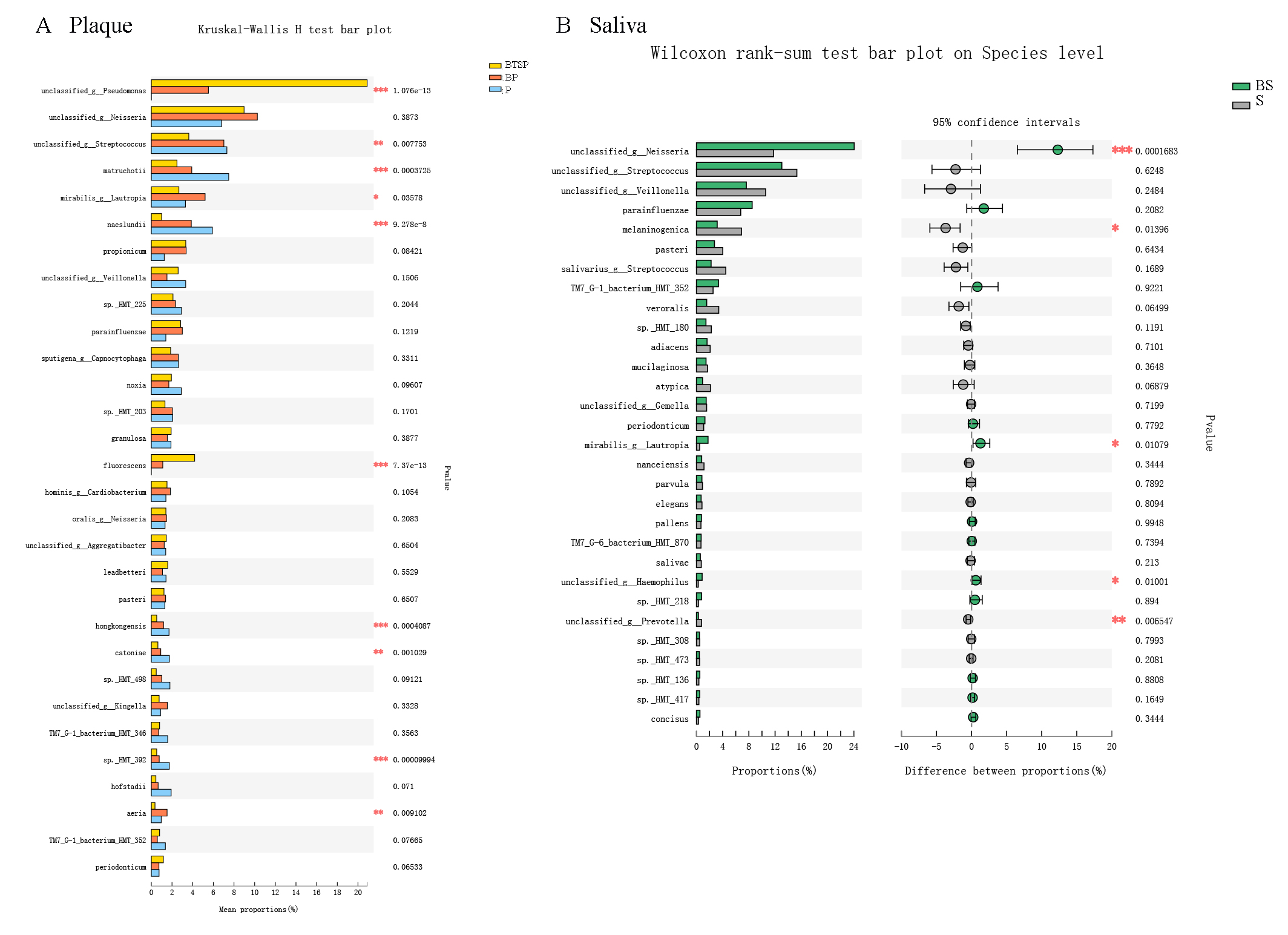


**Figure S3.** Comparisons of the relative abundance of bacterial communities. ^*^*P*<0.05, ^**^*P*<0.01, ^***^*P*<0.001. (A) Comparisons of the relative bacteria abundance among plaque groups at the species level using the Kruskal–Wallis H test. (B) Comparisons of the relative bacteria abundance in saliva from the black tooth stain and control groups at the species level using the Wilcoxon test.


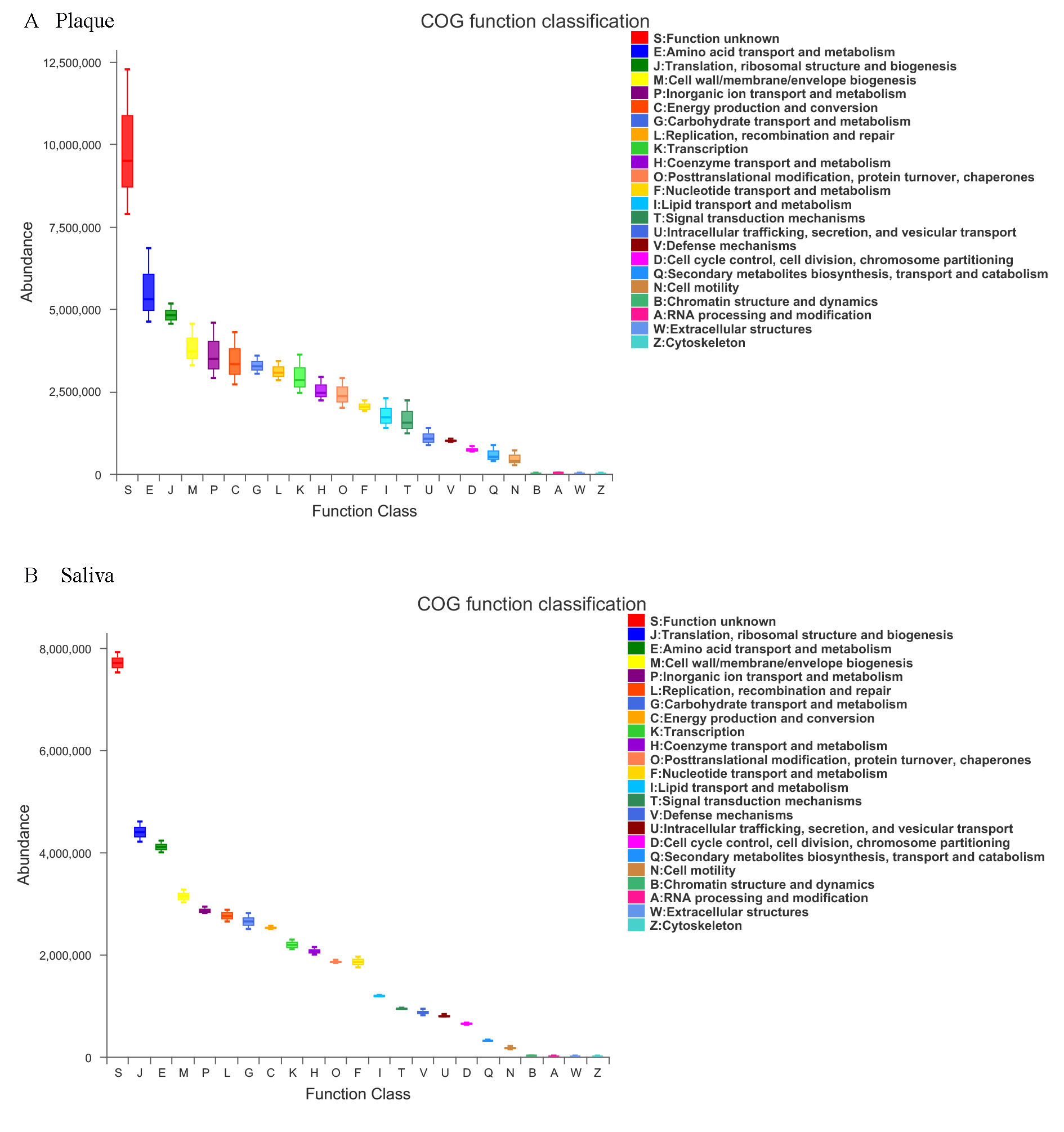


**Figure S4.** Functional annotation of the microbiome based on clusters of orthologous groups of proteins (COG); A) in dental plaque, B) in saliva.
